# Air in Wounds Innocuous

**Published:** 1871-01

**Authors:** 


Air in Wounds Innocuous.—Dr. Skey, of St. Bartholomew's (*Lancet*), repudiates the common view that the presence of air is injurious in wounds: "I remember hearing an eminent surgeon, when commenting on this commonly received opinion of the deleterious action of atmospheric air in wounds, assert that, had he undergone the operation for psoas abscess, he would have had the cavity of the abscess distended with air by a pair of bellows! In operations for empyema and hydrothorax, of which I have had a full share, I have never made any attempt to exclude air from the cavity of the chest; and I have published elsewhere one interesting case in which, in conjunction with the late Dr. Todd, we wilfully admitted sufficient air to fill a space previously occupied by six pints of serous fluid. This gentleman had not a bad symptom. In the largest example of emphysema from a broken rib I ever witnessed, the air distended the areolar tissue from the temple to the soles of the feet. It was all gradually removed—I suppose I must say absorbed—in the course of about ten days, except that contained in an enormously distended scrotum, which underwent no change in size, and exhibited no sign of mischief. This I punctured at the expiration of three weeks. A sudden gush of air, having no offensive character, escaped through the canula, and the scrotum fell flaccid; and in two or three days had recovered its ordinary aspect and sensibility."

